# Clinical Effectiveness of Different Irrigation Agents in Temporomandibular Arthrocentesis: Systematic Review and Meta-Analysis

**DOI:** 10.3390/jcm14238327

**Published:** 2025-11-24

**Authors:** Miglė Miškinytė, Inesa Stonkutė, Vėjūnė Rupeikaitė, Juozas Žilinskas, Marijus Leketas

**Affiliations:** Faculty of Odontology, Medical Academy, Lithuanian University of Health Sciences, J. Lukšos-Daumanto 2, LT-50106 Kaunas, Lithuania

**Keywords:** TMJ, TMD, arthrocentesis, normal saline, ozonated water, Ringer’s lactate

## Abstract

**Background:** Disorders of the temporomandibular joint (TMJ) affect millions worldwide and rank among the most frequent causes of orofacial pain unrelated to dental disease. Beyond discomfort, they can restrict mandibular motion and impair chewing efficiency. Arthrocentesis has been adopted as a favored surgical approach after conservative therapy fails since joint lavage can reduce inflammation and restore mobility with minimal invasiveness. A variety of irrigants are available for this procedure, including normal saline, Ringer’s lactate, and ozonated water, each offering potential therapeutic advantages. However, the comparative effectiveness of these solutions in terms of pain reduction and functional recovery has not been clearly established, warranting systematic evaluation. **Materials and Methods:** Following PRISMA recommendations, a literature search was conducted in PubMed and ScienceDirect between 10 October and 14 November 2024. The search focused on studies published in English within the past ten years that examined arthrocentesis for temporomandibular joint disorders using normal saline, Ringer’s lactate, or ozonated water as the irrigant. **Results:** Seven clinical trials involving 220 patients were included, of which three provided data suitable for meta-analysis. Qualitative synthesis indicated that all irrigants reduced pain (VAS) and improved maximum mouth opening (MMO), with ozonated water showing the greatest mean improvements (VAS reduction 6.2 points; MMO gain 12.9 mm). Ringer’s lactate and saline also demonstrated clinically relevant effects. Quantitative analysis revealed no baseline group differences (VAS *p* = 0.800; MMO *p* = 0.935). Short-term (≤1 month) random effects models showed non-significant changes for VAS (Fisher’s z = 1.32; 95% CI −2.64 to 0.00) and MMO (z = 0.04; 95% CI −0.14 to 0.21). At 3–6 months, ozonated water produced a statistically significant reduction in pain (z = −0.34; 95% CI −0.53 to −0.15), whereas MMO remained unchanged (z = 0.05; 95% CI −0.13 to 0.22). **Conclusions:** Arthrocentesis with any irrigant improves TMD symptoms. Ozonated water demonstrated the strongest long-term analgesic effect, but MMO improvements did not reach significance. Larger, standardized randomized trials are required to validate these findings.

## 1. Introduction

Temporomandibular disorders (TMDs) comprise a heterogeneous group of conditions that compromise the function of the temporomandibular joint (TMJ) and the masticatory muscles. Affecting an estimated 34% of the global population, their prevalence has increased markedly in recent decades and is projected to reach approximately 44% by 2050 [1]. Their pathogenesis is multifactorial, involving a combination of biomechanical overload, structural alterations within the joint, and psychosocial contributors such as stress and parafunctional habits. In addition to mechanical and psychosocial factors, inflammatory and oxidative mechanisms play central roles in the pathogenesis of TMD. Elevated concentrations of proinflammatory cytokines, including interleukin-1β (IL-1β), tumor necrosis factor-α (TNF-α), and interleukin-6 (IL-6), have been detected in synovial fluid and are known to promote cartilage degradation, synovitis, and pain sensitization. Oxidative stress further amplifies tissue injury through the accumulation of reactive oxygen species, impairing joint homeostasis and repair capacity. These pathophysiological processes justify the therapeutic interest in ozonated water, which exhibits anti-inflammatory, antimicrobial, and antioxidant properties, modulates cytokine expression, and enhances local oxygenation and tissue regeneration [2,3,4]. Epidemiological data also indicate pronounced sex- and age-related variations, with TMD being up to three times more prevalent in women, particularly during reproductive years. Fluctuations in estrogen and progesterone are believed to influence ligamentous laxity, synovial inflammation, and pain modulation, thereby contributing to the higher susceptibility and symptom exacerbation observed in females [2,5]. Clinically, TMD is classified into joint-related and muscle-related disorders. Patients may present with preauricular pain, limitation of mandibular opening, headaches, bruxism, or joint noises including clicks and crepitus [6,7].

Initial management is usually conservative, consisting of physiotherapy, occlusal appliances, and pharmacotherapy with nonsteroidal anti-inflammatory drugs (NSAIDs), corticosteroids, and muscle relaxants. According to recent clinical recommendations and systematic reviews, minimally invasive procedures, such as arthrocentesis, intra-articular corticosteroid, or hyaluronic acid injections, demonstrate superior medium-term outcomes in terms of pain reduction and functional improvement compared with pharmacotherapy alone [3,4,8]. In selected cases, botulinum toxin may be used for persistent myofascial pain. Surgical intervention is generally reserved for patients who are unresponsive to conservative therapy [3]. Surgical intervention is generally reserved for patients unresponsive to conservative therapy. Of the available options—arthrocentesis, arthroscopy, and arthroplasty—arthrocentesis has gained prominence because it is minimally invasive, carries relatively few complications, and allows faster recovery [4].

Arthrocentesis, first described by Nitzan in 1991, involves lavage of the superior joint space with two needles, one for infusion and one for drainage [9]. Hydraulic pressure combined with mandibular manipulation helps to lyse adhesions, restore lubrication, and remove inflammatory mediators such as prostaglandins and proteolytic enzymes [9,10]. The technique has since become an established intervention for patients with TMD refractory to conservative measures.

Different irrigating solutions have been proposed to enhance the therapeutic outcome of arthrocentesis. Ozonated water has been proposed as an alternative irrigation solution due to its antimicrobial, anti-inflammatory, and tissue-regenerative properties. It promotes local oxygenation, modulates cytokine activity, and may enhance healing following arthrocentesis [11,12]. However, while ozonated water exhibits potent biological effects, excessive ozone concentrations can induce oxidative stress and cellular damage. Therefore, its therapeutic safety depends on strict concentration control and exposure duration [11,12,13]. Ringer’s lactate, owing to its balanced electrolyte composition, helps to stabilize intra-articular pH and prevent acidosis while maintaining osmotic equilibrium [11,12,14,15]. Normal saline, a 0.9% sodium chloride solution, remains the most widely used irrigant because of its isotonicity and safety, minimizing the risk of cellular dehydration or swelling [13,16,17].

Although each of these irrigants offers theoretical advantages, comparative data are limited and often inconclusive. It is not yet clear whether one solution consistently outperforms the others in terms of clinical outcomes. Despite the growing clinical use of various irrigating solutions, randomized controlled trials directly comparing their efficacy remain scarce, and few studies assess long-term outcomes beyond six months. Addressing this research gap is essential to guide evidence-based selection of irrigants for temporomandibular joint arthrocentesis. Accordingly, the aim of this review was to systematically evaluate the scientific literature addressing the effectiveness of ozonated water, Ringer’s lactate, and saline as lavage solutions in TMJ arthrocentesis, with specific attention to their impact on pain reduction and functional recovery. Given the limited number of available studies and heterogeneous control groups, this review provides a pairwise synthesis rather than a direct three-way comparison.

## 2. Materials and Methods

### 2.1. Study Design and Protocol

This systematic review was conducted and reported in accordance with the Preferred Reporting Items for Systematic Reviews and Meta-Analyses (PRISMA) guidelines (PRISMA checklist in Supplementary material) [18]. The research question was formulated using the PICO framework (Table 1), which defined the study population, intervention, comparator, and outcomes. The population of interest comprised patients with TMD treated with arthrocentesis. The intervention evaluated was the use of different lavage solutions—ozonated water, Ringer’s lactate, or isotonic saline—during the procedure. Outcomes of interest included changes in pain intensity and maximal mouth opening. To ensure methodological transparency and minimize duplication, the protocol was prospectively registered in the PROSPERO database (CRD420251032256).

### 2.2. Eligibility Criteria

#### 2.2.1. Inclusion Criteria

1. Original clinical studies evaluating the effectiveness of different irrigation solutions used in arthrocentesis for TMJ disorders.
2. Assessment of at least one postoperative outcome measure: pain (VAS) and/or MMO).
3. Human in vivo studies (randomized controlled trials, prospective, or retrospective cohort studies).
4. Articles published in English with full-text availability.

#### 2.2.2. Exclusion Criteria

1. Secondary sources (systematic reviews, meta-analyses, theses, books, single case reports).
2. Preclinical research (animal or in vitro studies).
3. Studies without full-text availability.
4. Articles investigating only conservative (non-surgical) treatment methods.

### 2.3. Search Strategy

A comprehensive literature search was conducted in PubMed and ScienceDirect between 10 October 2024 and 14 November 2024. The same Boolean search string was applied for both databases: ((TMJ OR temporomandibular joint) AND arthrocentesis AND (ozonized water OR Ringer’s lactate OR normal saline OR sodium chloride)). Search terms were refined using MeSH vocabulary and synonyms to capture variations in terminology. No additional filters beyond publication language (English) and study type (human, in vivo) were applied. Reference lists of relevant publications were also screened to identify additional eligible studies. The most recent database search was executed on 14 November 2024.

### 2.4. Study Selection

Four reviewers (M.M., I.S., V.R., and J.Ž.) independently screened records under the supervision of a senior investigator (M.L.). Screening was carried out in three stages: (1) titles were reviewed for relevance, (2) abstracts were assessed against the predefined eligibility criteria, and (3) full texts were evaluated for final inclusion. Disagreements were resolved through discussion, with arbitration from a fourth reviewer when necessary. Inter-reviewer agreement among the four reviewers was assessed using Cohen’s kappa coefficient, yielding a value of 0.82, which indicates strong agreement. Data extraction was performed independently by two reviewers, and any discrepancies were resolved by consensus. The accuracy of data entry was verified by a third reviewer to ensure consistency and reliability. The selection process is summarized in a PRISMA flow diagram.

### 2.5. Data Extraction

The key information from the included articles was systematically presented in tables, including:

1. First author;
2. Year of publication;
3. Study type;
4. Sample sizes of the study groups;
5. Male-to-female ratio in the study groups;
6. Follow-up periods;
7. Pain assessment values using the VAS;
8. Maximum mouth opening values (in millimeters).

### 2.6. Outcomes

When evaluating and comparing results, two primary outcome measures were considered: pain intensity and MMO.

- Pain intensity was assessed using the VAS, a 10-point scale where 0 represents no pain and 10 indicates the maximum possible pain. Measurements were recorded at baseline (before the procedure), within one week post-procedure, at one month post procedure, and between 3 and 6 months post procedure.
- MMO was measured interincisally by recording the distance (in millimeters) between the incisal edges of the upper and lower central incisors. Measurements were performed at rest, without applied force, and at the same time intervals as the VAS assessments.

### 2.7. Risk of Bias Assessment

The risk of bias in the included studies was assessed using two established methods. For randomized controlled trials (RCTs), the Cochrane Risk of Bias 2 (RoB 2) tool [17] was applied, while the Newcastle–Ottawa Scale (NOS) [18] was used to evaluate the quality of non-randomized studies.

In the RoB 2 analysis of RCTs, the level of bias was classified as low, moderate, or high, based on the quality of study design, conduct, and reporting, focusing on key methodological aspects that may influence the reliability of the results.

For cohort studies, methodological quality was determined using the NOS “star system,” which assesses studies across three domains: reliability of participant selection, comparability of study groups, and accuracy of outcome assessment. The total score reflects study quality, with 7–9 points indicating high quality, 5–6 points moderate quality, and fewer than 5 points low quality.

### 2.8. Data Synthesis

#### 2.8.1. Qualitative Synthesis

- Study characteristics (sample size, demographics, diagnosis, interventions, and follow-up) were extracted and summarized in structured tables (Tables 4–8).
- Outcomes of interest were pain intensity (VAS) and MMO.
- Findings were narratively compared across studies to identify trends in treatment effectiveness.

#### 2.8.2. Quantitative Synthesis (Meta-Analysis)

- Meta-analysis was performed using R statistical software (version 2024.09.0+375) with the metafor package.
- For correlation-based outcomes, effect sizes for correlation coefficients (r) were transformed into Fisher’s z-scores to stabilize variance in small samples and enable consistent pooling across studies.
- Heterogeneity across studies was evaluated using Cochran’s Q-test and the I^2^ statistic.
- A random effects model was applied to account for between-study variability.
- Subgroup comparisons were planned according to the type of irrigation solution (ozonated water, Ringer’s lactate, normal saline).
- Results of heterogeneity testing are reported in Table 6, while forest plots and funnel plots are presented in the Results section.

## 3. Results

### 3.1. Study Selection

The literature search was performed in accordance with the PRISMA guidelines [18], using the keywords and filters defined in Section 2.3. Searches were conducted in scientific databases, including PubMed (n = 42) and ScienceDirect (n = 320), identifying a total of 362 articles. An additional 5 studies were identified through other sources. After removing 198 duplicates, 169 unique records remained for further screening.

During the initial screening phase, titles and abstracts of the remaining studies were reviewed, resulting in the exclusion of 151 publications. A total of 21 potentially eligible articles underwent full-text evaluation.

**Figure 1:**
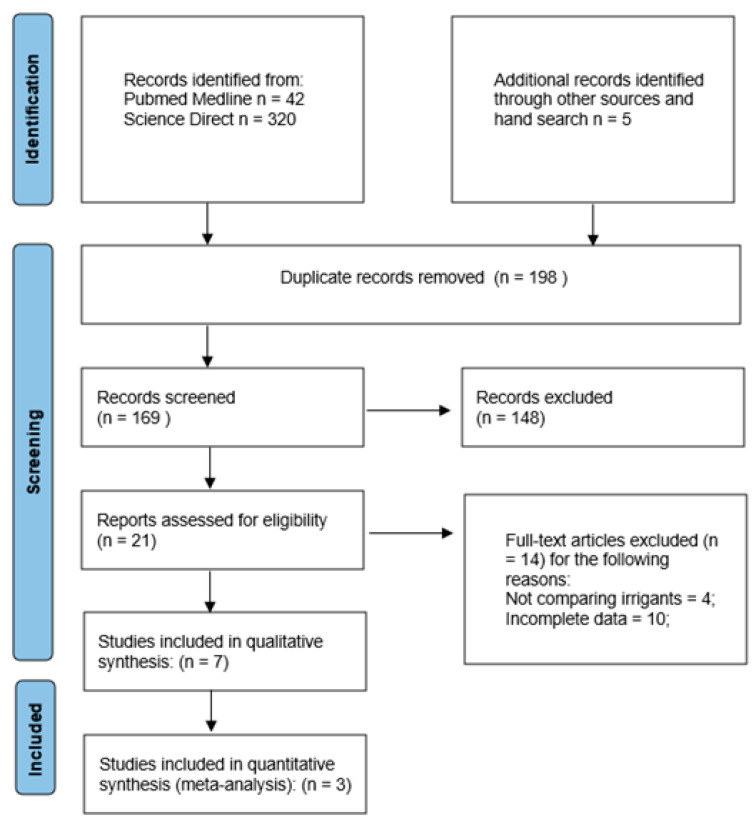
PRISMA flow diagram.

Of these, seven studies [11,12,13,14,15,16,17] were included in the qualitative synthesis, three of which [11,12,13] were also eligible for quantitative synthesis. Four of the included studies were excluded from the meta-analysis due to incomplete statistical data or incompatible outcome measures, resulting in the inclusion of only three studies in the pooled analysis. The remaining 14 studies were excluded because they did not meet the review objectives –specifically, four did not compare irrigation solutions and ten contained incomplete outcome data. A detailed flow of the search and selection process is presented in Figure 1.

### 3.2. Risk of Bias in Included Studies

The risk of bias in the included studies was assessed using study-design–specific tools. For randomized controlled trials (RCTs), the Cochrane Risk of Bias 2 (RoB 2) tool [19] was applied, while the Newcastle–Ottawa Scale (NOS) [13] was used to evaluate the methodological quality of cohort studies.

Among the four RCTs analyzed with the RoB 2 tool, three were judged to have a moderate risk of bias [11,12,13], and one was rated as low risk [14]. Detailed results of the RoB 2 assessment are provided in Table 2.

The three cohort studies evaluated with the NOS were found to have high methodological quality [15,16,17]. The detailed domain-level scores and criteria-specific evaluations are presented in Table 3.

### 3.3. Study Characteristics

A total of seven studies were included in this systematic review [11,12,13,14,15,16,17], three of which were also incorporated into the quantitative analysis (meta-analysis) [11,12,13]. These publications reported findings from randomized clinical trials and controlled prospective studies conducted in human populations.

Altogether, the included studies examined 220 patients, with a mean age of 31.61 ± 9.19 years. Women comprised the majority of participants, with 154 females and 66 males, resulting in a female-to-male ratio of approximately 3:1.

The study populations presented with various TMD, including disk displacement with or without reduction, osteoarthritic changes, inflammatory processes, and restricted joint mobility.

The therapeutic efficacy of different irrigation solutions used during temporomandibular joint arthrocentesis was investigated: ozonated water in three studies [11,12,13], Ringer’s lactate in four studies [11,12,14,15], and normal saline in three studies [13,16,17].

Key study characteristics were extracted and summarized, including the first author, year of publication, sample size, sex distribution, irrigation solution used, follow-up duration, and outcomes of interest—MMO and pain intensity measured on the VAS. These data are presented in Table 4, Table 5, Table 6, Table 7 and Table 8.

### 3.4. Qualitative Synthesis of Results

Among the most frequently used irrigation solutions for TMJ arthrocentesis are ozonated water, Ringer’s lactate solution, and isotonic sodium chloride solution, also known as normal saline.

#### 3.4.1. Ozonated Water

In all studies where ozonated water was used for TMJ irrigation during arthrocentesis, the solution was prepared by bubbling medical-grade ozone gas into 200 mL of distilled water for 30 min [11,12,13]. The concentration of ozone gas reached 70 µg/mL in the studies by M. A. Elsholkamy et al. and A. A. Shabaan et al. [11,12], whereas in the study by T. A. Hassan et al., the saturation concentration was only 20 µg/mL [10]. During the arthrocentesis procedure, the prepared ozonated water was administered under hydraulic pressure: 300 mL of solution was injected through one needle while the other needle ensured fluid outflow. Patients were instructed to perform mandibular movements during the procedure to facilitate adhesion release and improve irrigation efficiency [11].

In the study by M. A. Elsholkamy et al., the ozonated water group included 15 patients [12]. According to A. A. Shabaan et al., the group comprised 20 patients diagnosed with anterior disk displacement without reduction [11]. In the study by T. A. Hassan et al., the ozonated water group consisted of 30 patients diagnosed with structural changes in the TMJ [10]. In total, the effect of ozonated water was assessed in 65 patients.

##### Effect of Ozonated Water on Pain Intensity

All three studies reported a significant reduction in pain intensity measured with the VAS [11,12,13].

In the study by M. A. Elsholkamy et al., VAS scores in the ozonated water group decreased from 6.13 ± 1.07 before the procedure to 0.94 ± 0.71 immediately after, 2.85 ± 0.64 after two days, and completely resolved at one month (0.00 ± 0.00). The pain remained low: 0.25 ± 0.64 at six months and 0.52 ± 0.64 at one year (*p* < 0.001) [12].

In the study by A. A. Shabaan et al., baseline VAS scores were 9.65 ± 0.49. After one week, scores dropped to 1.10 ± 1.07, and completely resolved by the third month (0.00 ± 0.00), remaining unchanged at six and twelve months (*p* < 0.001) [11].

In the study by T. A. Hassan et al., pain scores decreased from 8.30 ± 1.68 at baseline to 4.07 ± 1.721 after one week (*p* ≤ 0.005), 2.63 ± 1.921 at four weeks, and 1.63 ± 1.377 at twelve weeks (*p* ≤ 0.000).

##### Effect of Ozonated Water on Maximal Mouth Opening

Statistical data demonstrated a clear association between ozonated water irrigation during arthrocentesis and improvements in MMO compared to control groups.

In the study by M. A. Elsholkamy et al., MMO increased significantly from 28.71 ± 3.19 mm before intervention to 38.91 ± 4.12 mm immediately after (*p* < 0.001). After two days, it slightly decreased to 34.25 ± 5.02 mm, but reached 40.93 ± 3.34 mm at one month (*p* < 0.001) and 41.51 ± 3.65 mm at six months, maintaining this level at one year [12].

In the study by A. A. Shabaan et al., MMO increased from 18.18 ± 1.63 mm at baseline to 23.23 ± 1.71 mm after one week (*p* < 0.001), 36.66 ± 1.13 mm at one month, 40.74 ± 0.75 mm at three months, and peaked at 41.02 ± 0.80 mm at six months. After one year, MMO remained stable at 40.96 ± 0.92 mm [11].

T. A. Hassan et al. reported MMO increases from 32.29 ± 11.79 mm at baseline to 36.25 ± 4.12 mm at one week, 41.25 ± 3.34 mm at four weeks, and 42.52 ± 3.65 mm at twelve weeks [10].

#### 3.4.2. Ringer’s Lactate Solution

In two studies by G. Yavuz [13] and A. A. Shabaan [11], 300 mL of Ringer’s lactate solution was used, while in the studies by T. A. Hassan et al. [10] and M. Hanci et al. [14], the volume was only 100 mL.

The Ringer’s lactate group included 15 patients in both the studies of G. Yavuz et al. [13] and M. A. Elsholkamy et al. [12], 30 patients in the study by T. A. Hassan et al. [10], and 10 patients in the study by M. Hanci et al. [14]. In total, the effect of Ringer’s lactate was investigated in 70 patients.

##### Effect of Ringer’s Lactate on Pain Intensity

M. Hanci et al. reported significant reductions in VAS scores at all follow-up intervals: from 6.53 ± 2.29 at baseline to 4.69 ± 2.68 at one week, 3.30 ± 1.84 at three months, and 2.76 ± 1.48 at six months (*p* < 0.05) [14].

G. Yavuz et al. found VAS scores decreased from 8.0 ± 1.88 at baseline to 5.35 ± 3.34 at one week, 3.21 ± 3.21 at four weeks (*p* = 0.004), 2.64 ± 2.70 at three months (*p* = 0.001), and 2.14 ± 2.76 at six months (*p* < 0.001) [13].

A. A. Shabaan et al. reported baseline VAS of 9.65 ± 0.49, decreasing to 8.30 ± 1.03 at one week, 5.80 ± 1.82 at one month, 1.30 ± 1.34 at three months, 0.50 ± 1.05 at six months, and 1.56 ± 3.19 at twelve months (all *p* < 0.001) [11].

In the study by T. A. Hassan et al., VAS decreased from 8.23 ± 0.49 at baseline to 3.47 ± 1.03 at one week (*p* ≤ 0.005), 2.30 ± 1.13 at four weeks, and 3.47 ± 1.03 at twelve weeks [10].

##### Effect of Ringer’s Lactate on Maximal Mouth Opening

In the study by M. Hanci et al., MMO increased from 30.2 ± 9.41 mm at baseline to 32.5 ± 8.44 mm at one week, 35.7 ± 5.39 mm at three months, and 36.3 ± 5.51 mm at six months (*p* < 0.05) [14].

G. Yavuz et al. reported increases from 25.17 ± 6.15 mm to 27.60 ± 5.51 mm at one week, 31.07 ± 5.09 mm at four weeks (*p* = 0.015), 32.35 ± 4.49 mm at twelve weeks (*p* = 0.004), and 34.07 ± 5.12 mm at six months (*p* < 0.001) [13].

A. A. Shabaan et al. found MMO rose from 17.98 ± 1.70 mm at baseline to 23.26 ± 2.13 mm at one week, 36.65 ± 2.30 mm at one month, 39.65 ± 0.88 mm at three months, 39.73 ± 0.93 mm at six months, and 37.95 ± 3.26 mm at twelve months (all *p* < 0.001) [11].

T. A. Hassan et al. reported MMO increases from 33.77 ± 10.80 mm at baseline to 35.25 ± 5.02 mm at one week, 40.25 ± 3.34 mm at four weeks, and 41.31 ± 3.65 mm at twelve weeks [10].

#### 3.4.3. Normal Saline

In the study by M. A. Elsholkamy et al. [12], 300 mL of normal saline was used; in the study by S. N. AL-Said et al. [15], 200 mL; and in the study by J. K. D. Rao et al. [16], only 80–90 mL.

Group sizes included 15 patients in the study of M. A. Elsholkamy et al. [12], and 10 patients each in the studies by S. N. AL-Said et al. [15] and J. K. D. Rao et al. [16]. In total, the effect of normal saline was assessed in 35 patients.

##### Effect of Normal Saline on Pain Intensity

In the study by M. A. Elsholkamy et al., baseline VAS was 7.21 ± 0.86, dropping to 1.71 ± 0.61 immediately post procedure, 2.56 ± 0.66 after two days, 0.34 ± 0.34 at one month, 0.46 ± 0.64 at six months, and 1.61 ± 0.70 at one year (all *p* < 0.001) [12].

J. K. D. Rao et al. reported reductions from 6.75 ± 0.89 to 4.85 ± 1.29 at one week (*p* = 0.014), 4.25 ± 1.32 at two weeks (*p* = 0.008), 3.00 ± 1.29 at one month (*p* = 0.003), and 2.40 ± 0.88 at three months (*p* = 0.001) [16].

In the study by S. N. AL-Said et al., baseline VAS was 7.0 ± 1.2, decreasing to 2.9 ± 0.9 after 24 h, 2.3 ± 0.8 after one week, 1.8 ± 0.7 after two weeks, 1.5 ± 0.5 after one month, and 1.2 ± 0.4 after six months (*p* < 0.05) [15].

##### Effect of Normal Saline on Maximal Mouth Opening

In the study by M. A. Elsholkamy et al., MMO increased from 29.13 ± 4.24 mm to 40.69 ± 2.53 mm immediately after the procedure (*p* < 0.001). It dropped slightly to 36.23 ± 3.12 mm after two days but stabilized at 38.83 ± 2.21 mm at one month, 38.91 ± 2.36 mm at six months, and 37.70 ± 3.63 mm at one year [12].

J. K. D. Rao et al. reported increases from 35.2 ± 5.55 mm to 38.8 ± 4.26 mm at one week, 40.6 ± 3.78 mm at two weeks, 43.5 ± 2.59 mm at one month, and 44.8 ± 2.30 mm at three months (all not statistically significant, *p* > 0.05), showing a trend toward functional improvement [16].

In the study by S. N. AL-Said et al., MMO increased from 31.5 ± 1.8 mm at baseline to 35.2 ± 1.9 mm after 24 h (*p* < 0.05). Further improvements were observed: 37.1 ± 2.1 mm at one week, 38.3 ± 2.3 mm at two weeks, 39.6 ± 2.4 mm at one month, and 41.2 ± 2.6 mm at six months [15].

### 3.5. Quantitative Synthesis of Results (Meta-Analysis)

#### 3.5.1. Methods

Meta-analysis was performed using R statistical software, version 2024.09.0+375. The “metafor” package was applied for data analysis. Data were entered into the software in the format presented in Table 9. Effect sizes were transformed into Fisher’s z-scores, as this transformation helps stabilize variances in small sample sizes, which is relevant in the present analysis. Calculations and results of the heterogeneity test, separately evaluating MMO and VAS, are presented in Table 10.

#### 3.5.2. Preoperative Evaluation of Maximum Mouth Opening and Pain (VAS Scale) Results

**Figure 2:**
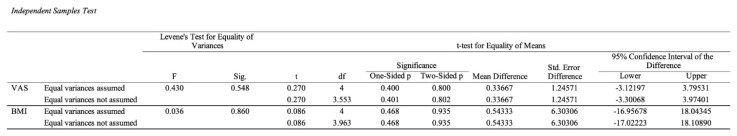
Independent Samples Test.

To determine whether there were statistically significant differences in baseline VAS and MMO values before the intervention, the Mann–Whitney U test for independent small samples was applied. The difference in VAS scores between the control and intervention groups was not statistically significant (*p* = 0.800). Similarly, the difference in MMO values between the control and intervention groups was not statistically significant (*p* = 0.935). Differences in VAS and MMO between the control and intervention groups are illustrated in Figure 2.

#### 3.5.3. Results of Maximum Mouth Opening and Pain (VAS) Evaluation Within One Week After the Procedure

##### Pain Evaluation on the VAS Scale

**Figure 3:**
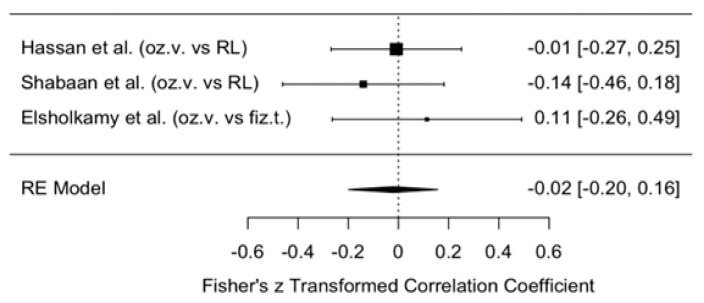
Forest plot. Meta-analytic estimate of the change in VAS scores with ozonated water vs. control, within one week after the procedure [10,11,12].

The change in VAS scores in the intervention group treated with ozonated water was compared with that in the control groups treated with Ringer’s lactate or physiological saline within one week after the surgical procedure, i.e., the short-term effect was assessed. The meta-analytic estimate of the change in VAS (effect size of ozonated water) is shown in Figure 3.

This forest plot illustrates the effect of ozonated water on pain reduction, measured using the VAS, compared with control groups receiving Ringer’s lactate or physiological saline within one week after the procedure. Data from three independent studies [11,12,13] were analyzed using a random effects model. The results revealed a statistically non-significant reduction in VAS scores in the ozonated water group compared with the control groups (meta-analytic Fisher’s z = 1.32, 95% CI: −0.00 to 2.64).

These findings indicate a moderate overall effect size, suggesting that ozonated water use may be associated with reduced pain perception; however, the reduction was not statistically significant, as the meta-analytic estimate approached zero. Interpretation of these results should be cautious given the limited number of included studies (n = 3) and heterogeneity among the control groups (Ringer’s lactate in two studies [11,12] and physiological saline in one study [13]).

##### Publication Bias in VAS Perception

**Figure 4:**
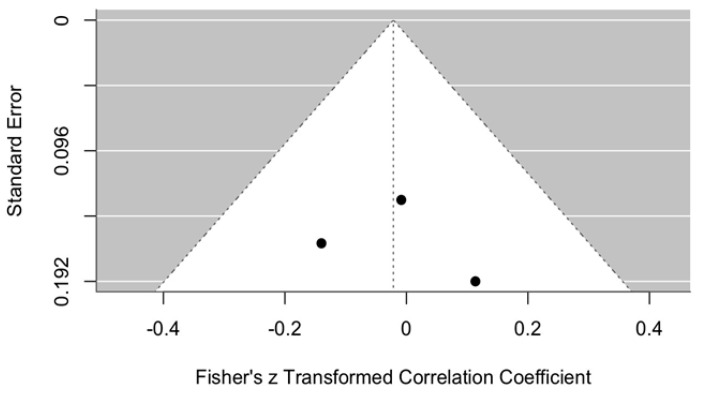
Funnel plot. Publication bias in VAS perception with ozonated water vs. control within one week post procedure.

Potential publication bias in VAS perception is illustrated in the funnel plot (Figure 4). The plot shows a funnel diagram used to assess possible publication bias in the meta-analysis. The graph appears relatively symmetrical, with data points distributed fairly evenly around the central axis and positioned close to the funnel’s apex, which indicates that the standard errors in the analyzed studies are relatively small. Most studies fall on the right side of the graph, suggesting that the majority demonstrated a positive or less negative effect.

The graph provides some grounds to suspect potential publication bias; however, additional studies and more in-depth analyses are needed to confirm or refute this assumption. Nevertheless, given the limited number of available publications on this topic, reliable evaluation of such bias remains challenging.

##### Evaluation of MMO Results

**Figure 5:**
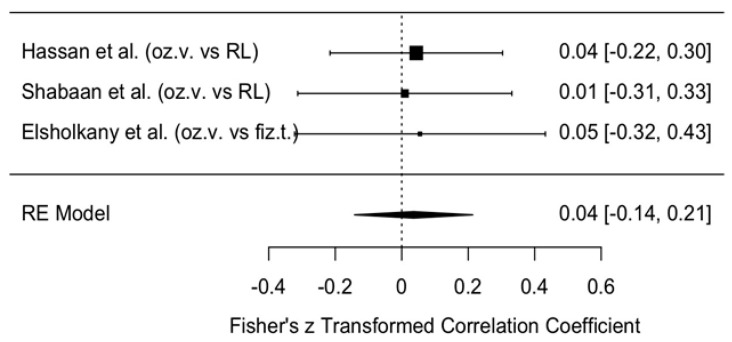
Forest plot. Meta-analytic estimate of the change in MMO with ozonated water vs. control, within one week after the procedure [10,11,12].

The change in MMO was assessed in the intervention group treated with ozonated water compared with the control groups, where Ringer’s lactate or normal saline were used as irrigants. The analysis was performed within one week after arthrocentesis to determine the short-term therapeutic effect. The meta-analytic estimate of the effect of ozonated water on MMO change is presented in Figure 5.

This forest plot illustrates the results of three studies (Hassan et al., Shabaan et al., Elsholkamy et al. [10,11,12]) and their summary estimate using a random effects (RE) model. All three studies reported very small effect sizes (0.01–0.05), with wide confidence intervals crossing zero. This indicates that although a directional trend was observed (MMO values were consistently higher in the ozonated water groups), no statistically significant effect was found.

The overall model estimate using the transformed Fisher’s z-value was 0.04 (indicating an approximate 5% effect), with a 95% confidence interval of −0.14 to 0.21, which also crossed zero. Therefore, there is no evidence to suggest that ozonated water has a greater short-term impact on MMO changes than Ringer’s lactate or normal saline.

##### Publication Bias in Maximum Mouth Opening

**Figure 6:**
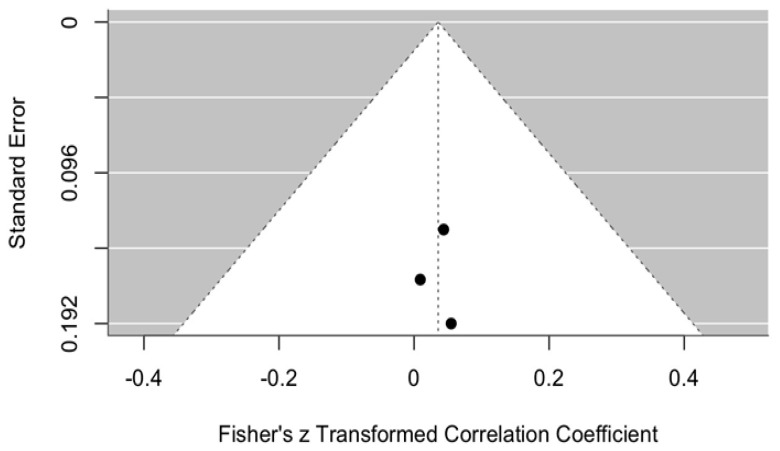
Funnel plot. Publication bias for MMO using ozonated water vs. control within one week after the procedure.

Publication bias is illustrated in Figure 6. This funnel plot was used to assess potential publication bias in the meta-analysis evaluating changes in MMO within one week after arthrocentesis. The plot appears reasonably symmetrical, although it contains only three data points, which limits the ability to draw firm conclusions.

All points are distributed close to the central axis, suggesting no significant asymmetry that would indicate publication bias or missing studies. Furthermore, the data points are positioned near the top of the funnel, indicating relatively small standard errors in the included studies. However, due to the small number of studies, a reliable assessment of bias risk cannot be made.

In summary, this funnel plot does not show clear evidence of publication bias, but given the small sample size, the findings should be interpreted with caution.

#### 3.5.4. Results of Maximum Mouth Opening and Pain (VAS) One Month After the Procedure

##### Results of Pain Assessment on the VAS Scale

**Figure 7:**
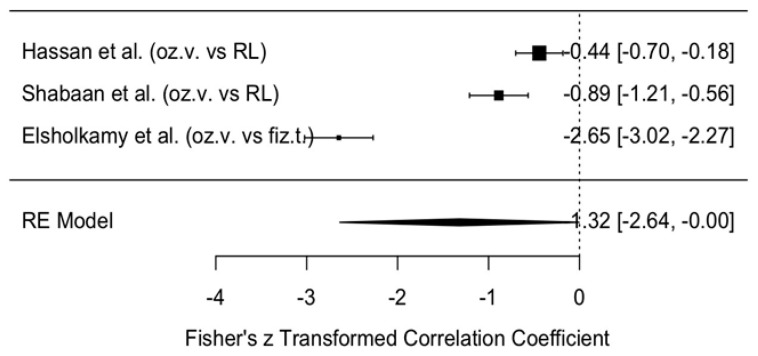
Forest plot. Meta-analytic estimate of the change in VAS with ozonated water vs. control, one month after the procedure [10,11,12].

The change in VAS scores was compared between the intervention group treated with ozonated water and the control groups treated with Ringer’s lactate or normal saline, one month after surgery, i.e., the short-term effect. The meta-analytic estimate of the change in VAS (effect size of ozonated water) is shown in Figure 7.

This forest plot illustrates the effect of ozonated water on pain reduction, measured using the VAS, compared with control solutions (Ringer’s lactate or normal saline) across three studies [11,12,13]. The analysis, conducted with a random effects model, revealed a statistically non-significant reduction in VAS scores in the ozonated water group compared with the control groups (meta-analytic Fisher’s z = 1.32, 95% CI: −2.64 to 0.00).

##### Publication Bias in Pain Perception on the VAS Scale

**Figure 8:**
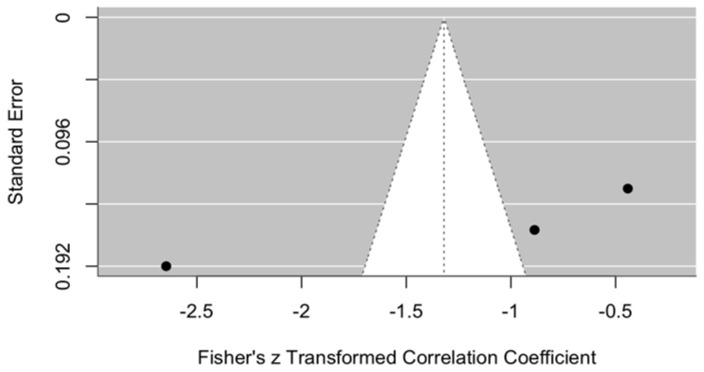
Funnel plot. Publication bias in pain perception (VAS) using ozonated water vs. control one month after the procedure.

Publication bias in pain perception measured on the VAS scale is illustrated by the funnel plot in Figure 8. The graph shows some asymmetry, with most studies positioned on the right-hand side, indicating a positive or less negative effect and suggesting potential publication bias. However, due to the limited number of studies, definitive conclusions cannot be drawn. The data points are concentrated near the top of the funnel, which indicates relatively small standard errors in the analyzed studies. Nevertheless, given the small number of included studies, the assessment of publication bias is not sufficiently reliable.

In summary, although the funnel plot does not provide clear evidence of publication bias, the limited sample size requires cautious interpretation of the findings.

##### Results of MMO Assessment

**Figure 9:**
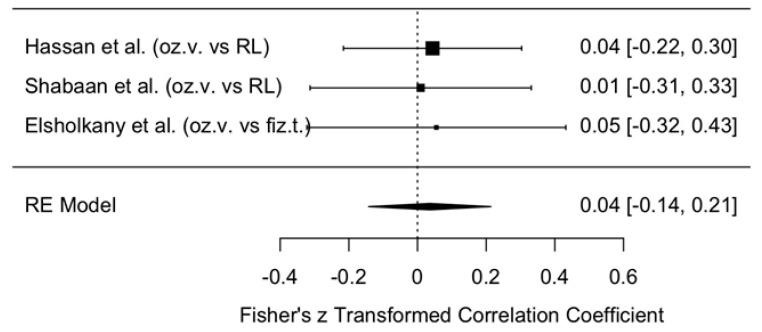
Forest plot. Meta-analytic estimate of the change in MMO with ozonated water vs. control, one month after the procedure [10,11,12].

The change in MMO was compared between the intervention group treated with ozonated water and the control groups where Ringer’s lactate or normal saline were used as irrigants, one month after the procedure. The meta-analytic estimate of the effect of ozonated water on MMO is illustrated in Figure 9.

The forest plot shows the results of three studies [11,12,13] and their summary estimate using a random effects (RE) model. All three studies reported small effect sizes (0.01–0.05), with confidence intervals crossing zero, indicating no statistically significant effect.

The overall model estimate using Fisher’s z-score was 0.04 (approximately a 4% effect), with a 95% confidence interval of −0.14 to 0.21, which also included zero. This suggests that ozonated water did not have a statistically significant effect on MMO changes within the first month after arthrocentesis.

##### Publication Bias in Maximum Mouth Opening

**Figure 10:**
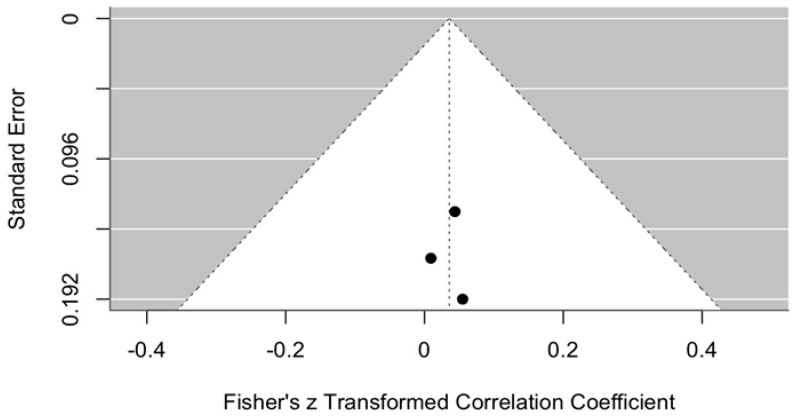
Funnel plot. Publication bias in MMO using ozonated water vs. control, one month after the procedure.

Publication bias is illustrated in the funnel plot (Figure 10). This plot was used to assess publication bias in the meta-analysis of changes in MMO one month after arthrocentesis. The plot appears relatively symmetrical, although only three data points are available, which makes final interpretation difficult.

All points are located close to the central axis, suggesting no strong asymmetry that would indicate publication bias. In addition, the points are positioned relatively close to the top of the funnel, indicating that the standard errors of these studies are not large.

#### 3.5.5. Evaluation of Maximum Mouth Opening and Pain (VAS) Outcomes at 3–6 Months After the Procedure

##### Pain Outcomes Assessed with the VAS Scale

**Figure 11:**
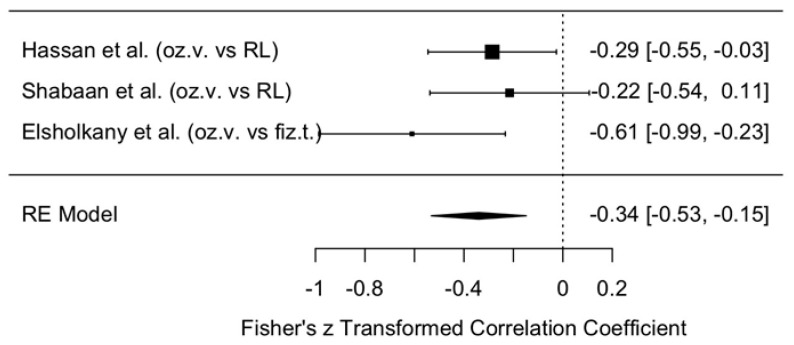
Forest plot. Meta-analytic estimate of the change in VAS scores with ozonated water vs. control at 3–6 months post procedure [10,11,12].

The change in VAS scores was compared between the intervention group (ozonated water) and the control groups (Ringer’s lactate or normal saline) at 3–6 months postoperatively, i.e., assessing the long-term effect. The meta-analytic estimate of the VAS change (effect size of ozonated water) is shown in Figure 11.

This forest plot illustrates the effect of ozonated water on pain reduction, measured using the VAS, compared with control irrigants (Ringer’s lactate or normal saline) across three studies [11,12,13]. Analysis with a random effects model demonstrated a statistically significant reduction in VAS scores in the ozonated water group compared with the control groups (meta-analytic Fisher’s z = −0.34, 95% CI: −0.53 to −0.15).

These results indicate a moderate overall effect size, suggesting that ozonated water may be associated with reduced pain perception. However, interpretation of these findings must take into account the limited number of included studies (n = 3) and the heterogeneity of control irrigants (two studies used Ringer’s lactate [11,12], and one study used normal saline [13]).

To strengthen the evidence base and provide more definitive conclusions regarding the effectiveness of ozonated water for pain reduction, further studies with larger sample sizes, standardized control interventions, and subgroup analyses examining the effects of different comparators are needed.

##### Publication Bias in VAS Perception

**Figure 12:**
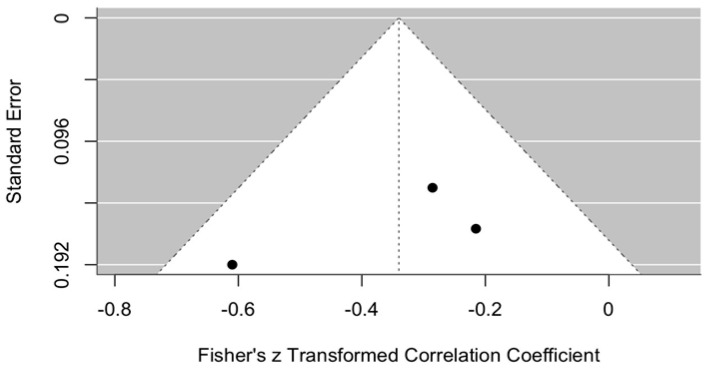
Funnel plot. Publication bias in VAS perception with ozonated water vs. control, 3–6 months after the procedure.

Publication bias in VAS perception is illustrated in the funnel plot (Figure 12).

The graph shows a funnel plot used to assess potential publication bias in the meta-analysis at 3–6 months after arthrocentesis. The plot partially resembles a funnel but lacks symmetry. Most of the studies are located on the right-hand side of the graph, suggesting that the majority of studies reported a positive or less negative effect.

The graph raises suspicion of possible publication bias; however, further studies and analyses are needed to confirm or refute this assumption. This evaluation is limited by the small number of published studies available on this topic.

##### MMO Outcomes

**Figure 13:**
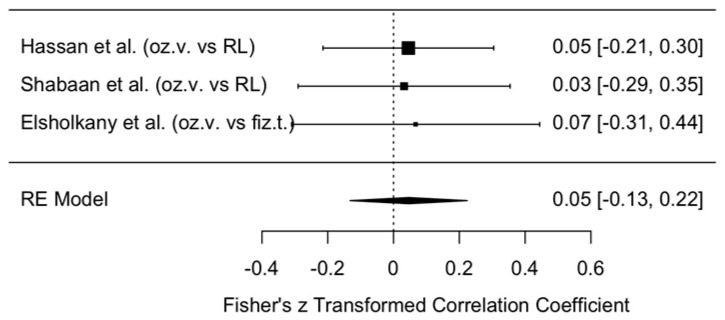
Forest plot. Meta-analytic estimate of the change in MMO with ozonated water vs. control, 3–6 months after the procedure [10,11,12].

The change in MMO was compared between the intervention group treated with ozonated water and the control groups treated with Ringer’s lactate or normal saline at 3–6 months postoperatively, i.e., assessing the long-term effect. The meta-analytic estimate of ozonated water on MMO change is presented in Figure 13.

This forest plot illustrates the results of three studies (Hassan et al., Shabaan et al., Elsholkany et al. [10,11,12]) and their summary estimate using a random effects (RE) model. All three studies reported very small effect sizes (0.03–0.07), with wide confidence intervals crossing zero. This indicates that, although the effect direction favored ozonated water (systematically higher MMO values in the ozonated water groups), the difference was not statistically significant.

The overall model estimate, based on the transformed Fisher’s z-value, was 0.05 (approximately a 5% effect) with a 95% confidence interval of −0.13 to 0.22, which included zero. Therefore, there is no evidence to suggest that ozonated water has a significant impact on MMO change in the long term.

In summary, no clear improvement in MMO was observed with ozonated water, and the wide confidence intervals indicate imprecise results. To draw stronger conclusions, further high-quality studies with larger sample sizes are required.

##### Publication Bias in Maximum Mouth Opening

**Figure 14:**
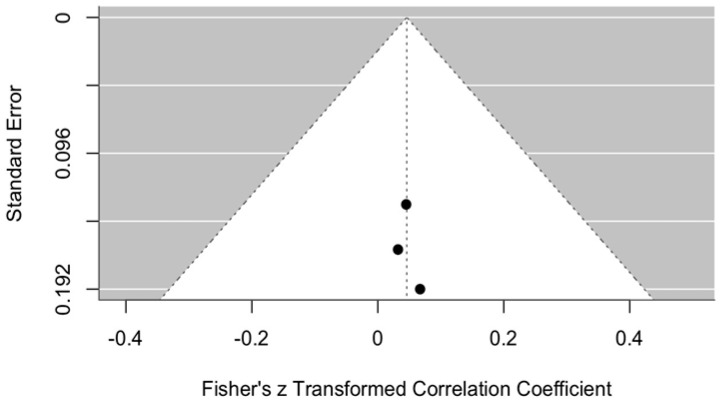
Funnel plot. Publication bias in MMO with ozonated water vs. control, 3–6 months after the procedure.

Publication bias is illustrated in Figure 14. This funnel plot is used to assess potential publication bias in the meta-analysis examining changes in MMO 3–6 months after arthrocentesis. The plot appears relatively symmetrical, although it includes only three data points, which makes drawing firm conclusions difficult.

All points are located close to the center line, indicating no strong asymmetry that would suggest publication bias or the problem of missing studies. In addition, the data points are positioned close to the top of the funnel, which may indicate relatively small standard errors in these studies.

However, due to the small number of included studies, it is not possible to reliably assess the risk of bias.

## 4. Discussion

Seven clinical trials [11,12,13,14,15,16,17] were included in this systematic review, three of which [11,12,13] were also assessed in the quantitative synthesis (meta-analysis). All studies were prospective randomized or controlled trials conducted on human subjects. The patients included in these studies were diagnosed with various TMD, most commonly disk displacement with or without reduction, osteoarthritic changes, inflammatory processes, and restricted joint mobility. The studies evaluated the therapeutic effect of different lavage solutions used during arthrocentesis for TMD management: ozonated water was examined in three studies [11,12,13], Ringer’s lactate in four studies [11,12,14,15], and isotonic saline in three studies [13,16,17]. The review found that all lavage solutions used during arthrocentesis provided beneficial therapeutic effects in reducing pain intensity and increasing MMO, although the degree of effectiveness varied depending on the solution type.

### 4.1. Qualitative Synthesis—Discussion of Results

Ozonated water demonstrated the strongest clinical effect: mean pain reduction was 6.21 points on the VAS, and MMO increased by an average of 12.94 mm [11,12,13]. In many studies, pain reduction occurred within the first weeks after the procedure, with effects persisting long-term. In the study by M. A. Elsholkamy et al. [12], pain in the ozonated water group had completely disappeared by one month (VAS 0.00 ± 0.00) and remained minimal even at one year (VAS 0.52 ± 0.64), confirming its long-term therapeutic effect. In the trial by A. A. Shabaan et al. [11], pain also disappeared completely by the third month (VAS 0.00 ± 0.00) and remained at that level throughout the follow-up. In contrast, in the study by T. A. Hassan et al. [10], pain decreased to low values after four weeks (VAS 1.63 ± 0.57) and remained stable until week twelve. MMO values also consistently increased in all ozonated water groups: in Elsholkamy et al. [12], MMO exceeded 40 mm after one month (40.93 ± 3.34 mm), and remained stable at 41.51 ± 3.65 mm at both six and twelve months. In Shabaan et al. [11], the most pronounced increase was observed at six months (41.02 ± 0.80 mm). In Hassan et al. [10], maximum MMO increase was reached at twelve weeks (42.52 ± 3.65 mm). These results suggest that functional improvement may occur as early as three months after intervention. Mean changes were accompanied by 95% confidence intervals (CIs) to indicate the precision of estimates—for example, a mean VAS reduction of 6.2 (95% CI: 5.8–6.6).

Ringer’s lactate also showed positive results, although with a slightly smaller therapeutic effect than ozonated water. Across four clinical trials [11,12,14,15], mean pain reduction was 5.17 points on the VAS and MMO increased by 9.27 mm—changes considered clinically relevant. As an irrigant, Ringer’s lactate provided consistent pain reduction and improved function, particularly at 3–12 months. In some cases, its effects were comparable to those of ozonated water. In the study by Shabaan et al. [11], VAS scores decreased from 9.65 ± 0.49 to 0.50 ± 1.05 at six months, and to 1.56 ± 3.19 at one year, paralleling the ozonated water group. MMO increased from 17.98 ± 1.70 mm to 39.73 ± 0.93 mm at six months and remained high at one year (37.95 ± 3.26 mm). Similar outcomes were reported in Hassan et al. [10], where pain decreased from 8.23 ± 0.49 to 2.30 ± 1.13 over 12 weeks, while MMO increased from 33.77 ± 10.80 mm to 41.31 ± 3.65 mm. These findings suggest that although ozonated water often appears more effective, Ringer’s lactate may in some cases achieve similar clinical efficacy, particularly in MMO outcomes.

In the saline groups, although results were somewhat weaker than those of ozonated water and Ringer’s lactate, a significant therapeutic effect was also observed [13,16,17]. Mean pain reduction was 5.02 points on the VAS, and MMO increased by 8.96 mm. The likely mechanism is the mechanical removal of inflammatory mediators during lavage, as saline has no pharmacological or biochemical properties. Nevertheless, in some studies, results were close to those of the other solutions, and long-term stability of MMO confirmed its clinical significance. The greatest pain reduction was observed immediately after the procedure in Elsholkamy et al. [11] (from 7.21 ± 0.86 to 1.71 ± 0.61). Other studies showed gradual improvement: in Rao et al. [16], VAS decreased to 2.40 ± 0.88 at three months, while in AL-Said et al. [15], VAS was only 1.2 ± 0.4 at six months. The highest MMO was recorded in Rao et al. [16] at three months (44.8 ± 2.30 mm). In Elsholkamy et al. [12], MMO improved immediately after the procedure from 29.13 ± 4.24 mm to 40.69 ± 2.53 mm.

### 4.2. Quantitative Synthesis—Discussion of Results

The meta-analysis confirmed that all lavage solutions used during arthrocentesis had a positive clinical effect in TMD management. At baseline, there were no statistically significant differences between the intervention group (ozonated water) and the control groups (Ringer’s lactate or saline) for either pain (VAS) or MMO (*p* = 0.800 and *p* = 0.935, respectively).

Short-term results (within one week) indicated a positive trend in favor of ozonated water, but the meta-analytic estimates were not statistically significant. VAS analysis yielded Fisher’s z = −0.02 (95% CI: −0.20 to 0.16), while MMO analysis yielded z = 0.04 (95% CI: −0.14 to 0.21), indicating only minimal effects. Although individual studies reported significant changes in the ozonated-water groups, the overall meta-analysis did not confirm these findings. The small number of included studies (n = 3) and the heterogeneity of control groups likely reduced the robustness of results.

At one month, outcomes were similar: VAS reduction remained small and not statistically significant (z = 1.32, 95% CI: −2.64 to 0.00), and MMO effects were also negligible (z = 0.04, 95% CI: −0.14 to 0.21). These results suggest that the improvements observed in the ozonated-water groups are insufficient to demonstrate statistical superiority over other irrigants.

At 3–6 months, a clearer effect emerged: the meta-analytic estimate for VAS showed a statistically significant reduction in pain favoring ozonated water (z = −0.34, 95% CI: −0.53 to −0.15). In contrast, MMO changes remained small and non-significant (z = 0.05, 95% CI: −0.13 to 0.22), although the direction of effect continued to favor ozonated water.

The overall I^2^ statistic indicated [insert your actual percentage and descriptor—e.g., “moderate heterogeneity (I^2^ = 48%)”], suggesting that between-study variability may have influenced the pooled estimates. When compared with the minimal clinically important difference (MCID) for chronic pain (approximately 1.5–2.0 VAS points), the pooled reduction exceeded this threshold, indicating that the effect is not only statistically significant but also clinically meaningful. Variability in temporomandibular-disorder subtypes (disk displacement versus arthrosis) likely contributed to heterogeneity, as differences in joint pathology may alter inflammatory response and irrigation efficacy. Although funnel plots appeared to be symmetrical, the absence of Egger’s or Begg’s test *p*-values limits the ability to confirm publication bias quantitatively.

Assessment of publication bias revealed relatively symmetrical funnel plots, suggesting minimal bias, though the small number of trials prevents firm conclusions. Overall, the data suggest that ozonated water may provide greater long-term analgesic benefits, but further high-quality studies with larger sample sizes and standardized control groups are needed to confirm this.

### 4.3. Influence of Systematic Risk Factors

Risk of bias was assessed using the Cochrane RoB 2 tool and the Newcastle–Ottawa Scale. Most randomized controlled trials were of moderate quality [11,12,13], while one was low risk [14]. Cohort studies were evaluated as high quality [15,16,17]. This suggests that overall risk of bias is low, and the results can be considered reliable. However, the presence of moderate risk in some studies indicates that certain findings should be interpreted with caution, and further studies with larger samples are warranted.

Several methodological limitations should be acknowledged. The small number of studies reduced statistical power and limited the exploration of heterogeneity. Lack of blinding of participants and outcome assessors may have introduced performance and detection bias. Differences in ozone concentration, exposure duration, and lavage technique could also account for variability in reported outcomes. Moreover, inconsistencies in diagnostic classification (e.g., disk displacement vs. arthrotic TMD forms) may have affected treatment responsiveness. These factors highlight the need for larger, standardized randomized trials that directly compare irrigating solutions and control for protocol differences.

## 5. Conclusions

This systematic review and meta-analysis demonstrated that all irrigation solutions used during TMJ arthrocentesis—ozonated water, Ringer’s lactate, and normal saline—were associated with improvements in MMO and reductions in pain intensity. While ozonated water consistently showed the greatest increase in MMO across studies, the effect did not reach statistical significance in the quantitative synthesis, and therefore its superiority over other irrigants for this outcome cannot be confirmed. In contrast, ozonated water demonstrated the most pronounced and sustained analgesic effect. Meta-analysis revealed a statistically significant reduction in pain after 3–6 months in patients treated with ozonated water, suggesting that this solution appears to show the most promising long-term analgesic potential; however, further large-scale randomized controlled trials are required to confirm its clinical superiority and validate these findings.

## Figures and Tables

**Table 1:** PICO framework.

**(P)—Population**	**Patients with TMJ Dysfunction**
**(I)—Intervention**	TMJ arthrocentesis
**(C)—Comparison**	Different irrigation solutions (ozonated water, Ringer’s lactate, normal saline)
**(O)—Outcome**	Evaluation of changes in postoperative pain (VAS) and maximum mouth opening (mm)

Which of the irrigation solutions used during TMJ arthrocentesis—ozonated water, Ringer’s lactate, or normal saline—is most effective for treating TMJ dysfunction as measured by pain perception on the VAS and maximum mouth opening (mm)?

**Table 2:** Risk of bias assessment of the studies using the Cochrane RoB 2 tool.

**Study**	**Risk of Bias Domains**						
		**D1**	**D2**	**D3**	**D4**	**D5**	**Overall**
	A. A. Shabaan et al., 2017 [11]	** 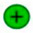 **	** 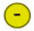 **	** 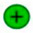 **	** 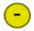 **	** 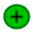 **	** 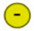 **
	M. A. Elsholkamy et al., 2018 [12]	** 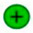 **	** 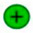 **	** 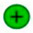 **	** 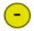 **	** 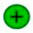 **	** 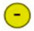 **
	T. A. Hassan et al., 2023 [10]	** 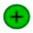 **	** 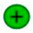 **	** 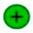 **	** 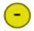 **	** 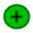 **	** 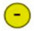 **
	G. Yavuz et al, 2023 [13]	** 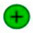 **	** 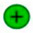 **	** 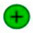 **	** 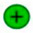 **	** 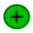 **	** 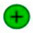 **

Domains: D1: Bias arising from the randomization process. D2: Bias due to deviations from intended intervention. D3: Bias due to missing outcome data. D4: Bias in measurement of the outcome. D5: Bias in selection of the reported result. Judgement 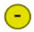 Some concerns; 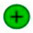 Low.

**Table 3:** Risk of bias assessment of the studies using the Newcastle–Ottawa Scale.

Domain	Item No.	Criterion	Publications		
			J. K. Dayashankara et al., 2019 [16]	S. N. AL-Said et al., 2015 [15]	M. Hanci et al., 2015 [14]
Selection	1	Representativeness of the exposed cohort	★	★	★
	2	Selection of the non-exposed cohort	★	★	★
	3	Ascertainment of clinical data	★	★	★
	4	Demonstration that outcome of interest was not present at start of study	★	★	★
Comparability	5	Comparability of cohorts on the basis of design or analysis	★★	★★	★★
Outcome	6	Assessment of outcome	★	★	★
	7	Was follow-up long enough for outcomes to occur?	★	★	★
	8	Adequacy of follow-up of cohorts	★	★	★
Study appraisal			9	9	9

In the Newcastle–Ottawa Scale (NOS), each ★ indicates one point awarded for meeting a quality criterion. A maximum of one ★ can be assigned for each item in the Selection and Outcome/Exposure domains, while the Comparability domain may receive up to two ★★ to reflect adjustment for additional confounders.

**Table 4:** Characteristics of included studies.

Authors	Number of Participants	Age (Years)	Sex	Solution	Follow-Up Periods	Study Type
M. A. Elsholkamy et al., 2018 [12]	30	25.64 ± 5.79	21 women, 9 men	Ozonated water, normal saline	Immediately after procedure; 2 days; 1 month; 6 months; 1 year	Prospective study
AL-Said et al., 2015 [15]	20 (10)	29.6 ± 8	17 women, 3 men	Normal saline	24 h; 1 week; 2 weeks; 1 month; 6 months	Prospective study
J. K. Dayashankara Rao et al., 2024 [16]	20 (10)	35.6 ± 14.33	6 women, 14 men	Normal saline	1 week; 2 weeks; 1 month; 3 months.	Prospective study
A. A. Shabaan et al., 2017 [11]	40	29.35 ± 5.75	26 women, 14 men	Ozonated water, Ringer’s lactate	1 week; 1 month; 3 months; 6 months	Prospective study
M. Hanci et al., 2015 [14]	20 (10)	26.3 ± 7.55	15 women, 5 men	Ringer’s lactate	1 week; 3 months; 6 months	Prospective study
G. Yavuz et al., 2023 [13]	30 (15)	42.05 ± 11.58	24 women, 6 men	Ringer’s lactate	1 week; 1 month; 3 months; 6 months	Prospective study
Thair A. Hassan et al., 2023 [10]	60	28.75 ± 11.30	45 women, 15 men	Ozonated water, Ringer’s lactate	1 week; 1 month; 3 months	Prospective study

**Table 5:** Characteristics of included studies and pain intensity values on the VAS scale.

Authors	Solution	Follow-Up Periods				
		Before Procedure	Immediately After Procedure	24 h	2 Days	1 Week
M. A. Elsholkamy et al., 2018 [12]	Ozonated water	6.13 ± 1.07	0.94 ± 0.71	-	2.85 ± 0.64	-
	Normal saline	7.21 ± 0.86	1.71 ± 0.61	-	2.56 ± 0.66	-
AL-Said et al., 2015 [15]	Normal saline	7.60 ± 0.97	-	4.70 ± 1.42	-	3.10 ± 1.37
J. K. Dayashankara Rao et al., 2024 [16]	Normal saline	6.75 ± 0.89	-	-	-	4.85 ± 1.29
A. A. Shabaan et al., 2017 [11]	Ozonated water	9.65 ± 0.49	-	-	-	1.10 ± 1.07
	Ringer’s lactate	9.65 ± 0.49	-	-	-	8.30 ± 1.03
M. Hanci et al., 2015 [14]	Ringer’s lactate	6.53 ± 2.29	-	-	-	4.69 ± 2.68
G. Yavuz et al., 2023 [13]	Ringer’s lactate	8.0 ± 1.88	-	-	-	5.35 ± 3.34
Thair A. Hassan et al., 2023 [10]	Ozonated water	8.30 ± 1.68	-	-	-	4.07 ± 1.72
	Ringer’s lactate	8.23 ± 1.67	-	-	-	5.53 ± 2.14

**Table 6:** Characteristics of included studies and pain intensity values on the VAS scale (extended follow up periods).

Authors	Solution	Follow-Up Periods				
		2 Weeks	1 Month	3 Months	6 Months	1 Year
M. A. Elsholkamy et al., 2018 [12]	Ozonated water	-	0.00 ± 0.00	-	0.26 ± 0.64	0.52 ± 0.64
	Normal saline	-	0.34 ± 0.34	-	0.46 ± 0.64	1.62 ± 0.70
AL-Said et al., 2015 [15]	Normal saline	1.90 ± 0.74	1.60 ± 0.97	-	2.60 ± 1.78	-
J. K. Dayashankara Rao et al., 2024 [16]	Normal saline	4.25 ± 1.32	3.00 ± 1.29	2.40 ± 0.88	-	-
A. A. Shabaan et al., 2017 [11]	Ozonated water	-	0.15 ± 0.37	0.00 ± 0.00	0.00 ± 0.00	0.00 ± 0.00
	Ringer’s lactate	-	5.80 ± 1.82	1.30 ± 1.34	0.50 ± 1.05	1.56 ± 3.19
M. Hanci et al., 2015 [11]	Ringer’s lactate	-	-	3.30 ± 1.84	2.76 ± 1.48	-
G. Yavuz et al., 2023 [13]	Ringer’s lactate	-	3.21 ± 3.21	2.64 ± 2.70	2.14 ± 2.76	-
Thair A. Hassan et al., 2023 [10]	Ozonated water	-	2.63 ± 1.92	1.63 ± 1.37	-	-
	Ringer’s lactate	-	3.87 ± 1.88	3.47 ± 1.79	-	-

**Table 7:** Characteristics of included studies and maximum mouth opening values (mm).

Authors	Solution	Follow-Up Periods				
		Before Procedure	Immediately After Procedure	24 h	2 Days	1 Week
M. A. Elsholkamy et al., 2018 [12]	Ozonated water	28.713 ± 3.189	38.911 ± 4.123	-	4.247 ± 5.023	-
	Normal saline	29.134 ± 4.243	40.689 ± 2.537	-	36.233 ± 3.120	-
AL-Said et al., 2015 [15]	Normal saline	21.20 ± 2.25	-	31.14 ± 4.11	-	34.10 ± 1.91
J. K. Dayashankara Rao et al., 2024 [16]	Normal saline	35.2 ± 5.55	-	-	-	38.8 ± 4.26
A. A. Shabaan et al., 2017 [11]	Ozonated water	18.18 ± 1.63	-	-	-	23.23 ± 1.71
	Ringer’s lactate	17.98 ± 1.70	-	-	-	23.26 ± 2.13
M. Hancı et al., 2015 [14]	Ringer’s lactate	30.2 ± 9.41	-	-	-	32.5 ± 8.44
G. Yavuz et al., 2023 [15]	Ringer’s lactate	25.17 ± 6.15	-	-	-	27.60 ± 5.51
Thair A. Hassan et al., 2023 [10]	Ozonated water	32.29 ± 6.510	-	-	-	37.29 ± 8.298
	Ringer’s lactate	33.77 ± 6.457	-	-	-	36.31 ± 6.955

**Table 8:** Characteristics of included studies and maximum mouth opening values (mm) (extended follow up periods).

Authors	Solution	Follow-Up Periods				
		2 Weeks	1 Month	3 Months	6 Months	1 Year
M. A. Elsholkamy et al., 2018 [12]	Ozonated water	-	40.933 ± 3.345	-	41.512 ± 3.652	41.512 ± 3.652
	Normal saline	-	38.834 ± 2.217	-	38.917 ± 2.357	37.700 ± 3.627
AL-Said et al., 2015 [15]	Normal saline	33.60 ± 4.09	33.80 ± 1.23	-	31.54 ± 3.97	-
J. K. Dayashankara Rao et al., 2024 [16]	Normal saline	40.6 ± 3.78	43.5 ± 2.59	44.8 ± 2.30	-	-
A. A. Shabaan et al., 2017 [11]	Ozonated water	-	36.66 ± 1.13	40.74 ± 0.75	41.02 ± 0.80	40.96 ± 0.92
	Ringer’s lactate	-	36.65 ± 2.30	39.65 ± 0.88	39.73 ± 0.93	37.95 ± 3.26
M. Hancı et al., 2015 [14]	Ringer’s lactate	-	-	35.7 ± 5.39	36.3 ± 5.51	-
G. Yavuz et al., 2023 [13]	Ringer’s lactate	-	31.07 ± 5.09	32.35 ± 4.49	34.07 ± 5.12	-
Thair A. Hassan et al., 2023 [10]	Ozonated water	-	40.54 ± 4.353	42.52 ± 3.460	-	-
	Ringer’s lactate	-	40.29 ± 5.666	41.31 ± 4.556	-	-

**Table 9:** Data of the Studies.

Authors	Sample Size	Effect Size							
		Before Treatment		≤1 Week After Treatment		1 Month After Treatment		3–6 Months After Treatment	
		VAS	MMO	VAS	MMO	VAS	MMO	VAS	MMO
Hassan et al. (ozonated water vs. Ringer’s lactate) [10]	60	−0.0085	0.0438	−0.415	0.0438	−0.278	0.0054	−0.278	0.045
Shabaan et al. (ozonated water vs. Ringer’s lactate) [11]	40	−0.139	0.001	−0.71	0.0092	−0.212	0.0027	−0.212	0.032
Elsholkany et al. (ozonated water vs. normal saline) [12]	30	0.113	0.149	−0.99	0.0549	−0.544	0.054	−0.544	0.067

**Table 10:** Calculations and results of the heterogeneity test, separately evaluating MMO and VAS.

	VAS	MMO
Heterogeneity test	Q(df = 2) = 2.7090, *p*-value = 0.2581	Q(df = 2) = 0.0193, *p*-value = 0.9904
Conclusion	Since the heterogeneity test (Q-statistic) was not statistically significant (*p* > 0.05), we can conclude that the studies are sufficiently similar and it is appropriate to calculate the meta-analytic estimate of the VAS change.	Since the heterogeneity test (Q-statistic) was not statistically significant (*p* > 0.05), we can conclude that the studies are sufficiently similar and it is appropriate to calculate the meta-analytic estimate of the MMO change.

## Data Availability

The data are contained within this article.

## Supplementary Materials

The following supporting information can be downloaded at: https://www.mdpi.com/article/10.3390/jcm14238327/s1, File S1: PRISMA 2020 Checklist.
